# Supplementary material for: Interactions of nuclear transport factors and surface-conjugated FG nucleoporins: Insights and limitations
Source: PLoS One. 2019 Jun 6;14(6):e0217897. doi: 10.1371/journal.pone.0217897 (PMC6553764; doi:10.1371/journal.pone.0217897)
Supplement: S1 Text — (PDF) [file pone.0217897.s001.pdf]

S1 Text. Primary sequences of the protein constructs.

Nsp1FG-Cys-His$_6$

MSTGAGAFGTGQSTFGFNNSAPNNTNNANSSITPAFGSNNTGNTAFGNSNPTSNVFGSNNSTTNT
FGSNSAGTSLFGSSSAQQTKSNGTAGGNTFGSSSLFNNSTNSNTTKPAFGGLNFGGGNNTTPSST
GNANTSNNLFGATANANKPAFSFGATTNDDKKTEPDKPAFSFNSSVGNKTDAQAPTTGFSFGSQ
LGGNKTVNEAAKPSLSFGSGSAGANPAGASQPEPTTNEPAKPALSFGTATSDNKTTNTTPSFSFG
AKSDENKAGATSKPAFSFGAKPEEKKDDNSSKPAFSFGAKSNEDKQDGTAKPAFSFGAKPAEKN
NNETSKPAFSFGAKSDEKKDGDASKPAFSFGAKPDENKASATSKPAFSFGAKPEEKKDDNSSKPA
FSFGAKSNEDKQDGTAKPAFSFGAKPAEKNNNETSKPAFSFGAKSDEKKDGDASKPAFSFGAKS
DEKKDSDSSKPAFSFGTKSNEKKDSGSSKPAFSFGAKPDEKKNDEVSKPAFSFGAKANEKKESDE
SKSAFSFGSKPTGKEEGDGAKAAISFGAKPEEQKSSDTSKPAFTFGCLEHHHHHH*

FSFG$_6$-Cys-His$_6$

MNETSKPAFSFGAKSDEKKDGDASKPAFSFGAKPDENKASATSKPAFSFGAKPEEKKDDNSSKP
AFSFGAKSNEDKQDGTAKPAFSFGAKPAEKNNNETSKPAFSFGAKSDEKKDGDASKPACLEHHH
HHH*

SSSG$_6$-Cys-His$_6$

MNETSKPASSSGAKSDEKKDGDASKPASSSGAKPDENKASATSKPASSSGAKPEEKKDDNSSKP
ASSSGAKSNEDKQDGTAKPASSSGAKPAEKNNNETSKPASSSGAKSDEKKDGDASKPACLEHHH
HHH*
