# Supplementary material for: Interactions of nuclear transport factors and surface-conjugated FG nucleoporins: Insights and limitations
Source: PLoS One. 2019 Jun 6;14(6):e0217897. doi: 10.1371/journal.pone.0217897 (PMC6553764; doi:10.1371/journal.pone.0217897)
Supplement: S2 Text — (PDF) [file pone.0217897.s002.pdf]

S2 Text. Estimating the impact of the mixing lag on the kinetic parameters in QCM-D experiments.

The QCM-D results in Fig 3B clearly indicate that mass transport per unit time was limiting at lower flow rates in Kap95 binding experiments. It is interesting to note that when re-plotting the data as ΔF (a proxy for mass deposited) versus the total volume passed (a proxy for the moles of Kap95 passed over the surface), the binding curves at different flow rates become superimposable with each other (S1A-B Fig), indicating that the *binding capacity* of the FG Nup layer (which we will denote as ΔF_{max}) is not affected by the flow rate. Hence the equilibrium for the particular reaction of interest should not be affected by the flow rate either; however, we did not conduct an equilibrium analysis because we could not confirm if the system reached equilibrium in our experiments as discussed in the main text.

In contrast, an estimation of the kinetic parameters is affected by the flow rate (S1C-D Fig). Assuming a simple Langmuir model, the binding equation can be expressed as:

$$\frac{d\Delta F}{dt} = k_{on}C_{actual}(\Delta F_{max} - \Delta F) - k_{off}\Delta F \quad (1),$$

where k_{on} and k_{off} are the binding and unbinding rate constants, respectively. C_{actual} is the actual concentration of the analyte within the QCM-D chamber. At $t \sim 0$ s, it is reasonable to assume $\Delta F \sim 0$ Hz^{-1}, hence the equation (1) can be simplified as:

$$\frac{d\Delta F}{dt} = k_{on}C_{actual}\Delta F_{max} \quad (2).$$

If we neglect the mass transport limitation (MTL), i.e. if we assume that the sensing volume is instantaneously replaced with the feed solution, we can assume C_{actual} to be equal to C_{feed}, the concentration of the analyte in the feed solution. Then the equation (2) can be re-written as:

$$\frac{\Delta F(t)}{\Delta t} = k_{on}C_{feed}\Delta F_{max} \quad (3)$$

We fitted this equation to the early time points in the QCM-D binding data to examine whether the rate of binding is dependent on the flow rate. The left-hand side of the equation (3), the rate of change in ΔF per unit time, in the early time points varied with the flow rate (S1D Fig); absolute values of these rates increased with the flow rate. ΔF_{max} in the right-hand side of the equation is independent of the flow rate (see above), so the remaining term on the right-hand side, $k_{on}C_{feed}$, would be dependent on the flow rate. According to the volume replacement experiment with glycerol (S1E-G Fig), it is evident that C_{actual} does not reach C_{feed} instantaneously, so using the equation (3) to calculate k_{on} would underestimate its value.

For example, at the slowest flow rate used in our experiments, 50 µl/min, the mixing lag was ~4 min (S1E Fig). Consequently, C_{actual} is not equal to C_{feed} during the first 0.4 min upon the introduction of analytes, a period in which the linear approximation can be made (S1D Fig). Instead, $C_{actual} = 0.025 \cdot C_{feed}$ (S1G Fig). To simplify this analysis, one can use an average analyte concentration between time 0 min to 0.4 min to assess how the estimation of k_{on} would be different with or without taking MTL into consideration:

$$k_{on,no\ MTL} C_{feed} \Delta F_{max} = k_{on,actual} \left(\frac{0 + 0.025}{2} C_{feed} \right) \Delta F_{max} \quad (4),$$

where the left-hand side assumes no MTL and the right-hand side takes the average of the C_{actual} at the initial and the end points to take into account the mixing lag. By simplifying the equation, we get:

$$k_{on,no\ MTL} \sim 0.01\ k_{on,actual} \quad (5),$$

indicating that k_{on} calculated without taking into consideration the MTL ($k_{on,no\ MTL}$) would be two orders of magnitudes lower than what it should be ($k_{on,actual}$). The same effect was present even at 300 µl/min, the flow rate used for the binding experiments. At this flow rate, C_{actual} was equal to $0.4 \cdot C_{feed}$ after 0.4 min (S1G Fig); neglecting MTL would underestimate the k_{on} by at least ~5-fold at 300 µl/min.

We also note that this simple analysis assumes C_{actual} to be constant during the time period of interest, though in reality it is a function of time in the binding experiments. A fraction of analytes will be adsorbed onto the surface, and thus, C_{actual} would be dependent on factors that vary with time; C_{actual} is even lower than the one calculated in this simple analysis, and so is the extent underestimation of k_{on} value. Furthermore, when taking into account other factors discussed throughout the text, such as multivalency of the interaction, it would be much difficult to assess the effect of flow rate on the kinetic parameters, because a simple Langmuir model will not be appropriate and the MTL will be interwound with other complex reaction mechanisms.
