# Supplementary material for: Interactions of nuclear transport factors and surface-conjugated FG nucleoporins: Insights and limitations
Source: PLoS One. 2019 Jun 6;14(6):e0217897. doi: 10.1371/journal.pone.0217897 (PMC6553764; doi:10.1371/journal.pone.0217897)
Supplement: S3 Text — (PDF) [file pone.0217897.s003.pdf]

S3 Text. SPR - Simulating simple protein-protein interactions by multiphysics finite element model.

To test the prevalence of mass transport limitation, we conducted numerical simulations on hypothetical protein-protein interactions in a simulated SPR chamber. For these simulations, we modeled a set of proteins with lower molecular weights than those of the FG constructs and Kap95 used in our experiments. These lower molecular weight proteins diffuse faster and should thus provide useful lower limits on the magnitude of mass transport limitations in our experiments. To numerically simulate such interactions, a multiphysics finite element model was developed to understand the role of convection mass transport in a particular geometry of the SPR chamber used in the experiment. The SPR chamber was set to be a rectangular prism 1 mm deep, 1 mm wide, and 0.5 mm high. The surface ligand density, molecular weight of the ligand, molecular weight and concentration of the analyte, and finally binding site kinetics used in the model are summarized in Table S2. The model considered several aspects of the system. The volumetric flow rate of 10, 100, and 1,000 µl/min were used as the inlet boundary condition for the flow, which all resulted in laminar flow field. Transport through the SPR system was modeled with the following system of equations and boundary conditions, assuming the analyte solution was dilute:

$$\frac{dc_i}{dt} + \nabla \cdot (-D_i \nabla c_i) + \boldsymbol{u} \cdot \nabla c_i = R_i$$

$$\boldsymbol{N}_i = -D_i \nabla c_i + \boldsymbol{u} c_i$$

Boundary condition at the wall:	$-\boldsymbol{n} \cdot \boldsymbol{N}_i = 0$
Boundary condition at the inlet:	$\boldsymbol{n} \cdot \boldsymbol{N}_i = \boldsymbol{n} \cdot (\boldsymbol{u} c_{0,i})$
Boundary condition at the outlet:	$-\boldsymbol{n} \cdot D_i \nabla c_i = 0$

where c_i is the concentration of species *i*, $c_{0,i}$ is the concentration of species *i* at the inlet (i.e., feed solution concentration), D_i the diffusivity of species *i*, $\boldsymbol{u}$ the velocity, R_i the source term representing the net rate of formation of species *i* per unit volume by chemical reaction (i.e. binding/unbinding using Langmuir model), N_i the molar flux of species *i*. The diffusivity of the analyte molecules themselves, $D_{analyte}$, were calculated from their molecular weights using Polson equation:

$$D_{analyte} = \frac{9.40 * 10^{-15} * T}{\mu * \sqrt[3]{MW_{protein}}}$$

where *T* is the temperature, μ the solution viscosity, and *MW* the molecular weight of the analyte. As shown by the equations above, transport of analytes in the bulk solution was coupled with a surface reaction, R_i, on the lower surface of the SPR chamber that represented analyte binding to its ligand, using a Langmuir binding model.

In a simulation, the SPR chamber was devoid of analytes initially and the solution containing the analyte was introduced into the chamber at $t = 0$ s. Transient flow simulations were run until the outlet concentration of the analyte reached a steady-state value equal to the inlet concentration. The outlet analyte concentration (averaged over the exit 1 mm x 0.5 mm surface area) and bound surface analyte amount (bound on the lower 1 mm x 1 mm SPR reaction area) were charted to see how convection, diffusion, and the binding reaction all interact to govern the surface binding in the SPR instrument.
