# Supplementary material for: Interactions of nuclear transport factors and surface-conjugated FG nucleoporins: Insights and limitations
Source: PLoS One. 2019 Jun 6;14(6):e0217897. doi: 10.1371/journal.pone.0217897 (PMC6553764; doi:10.1371/journal.pone.0217897)
Supplement: S1 Fig — (PDF) [file pone.0217897.s004.pdf]

S1 Fig. Estimating the impact of the mixing lag on the kinetic parameters in QCM-D experiments.

(A-D) The dependence of Kap95 binding to Nsp1FG surface on the flow rate. The same QCM-D data as in Fig 3B were used here. (A) ΔF_5 at different flow rates were plotted against the total volume passed through. The grey box indicates the regions magnified in (B). (C) A replicate of Fig 3B. (D) Magnification of the first 0.4 min shown in (C). (E-G) Estimating the mixing lag (t_{lag}) based on the volume replacement experiment shown in Fig 3C. (E) Estimate of t_{lag} at different flow rates. t_{lag} at each flow rate was calculated as the time it takes to replace 99% of the sensing volume. Volume replacement was tracked by the change in ΔF_5 with $\Delta F_5 = 0$ Hz as the initial point and the final asymptotic line (ΔF_5 = ~-15 Hz) as the end point. (F) The data show in Fig 3C replotted after normalization by ΔF_5 at the final asymptotic line. The early time points (up to 1 min) in (F) are magnified in (G).

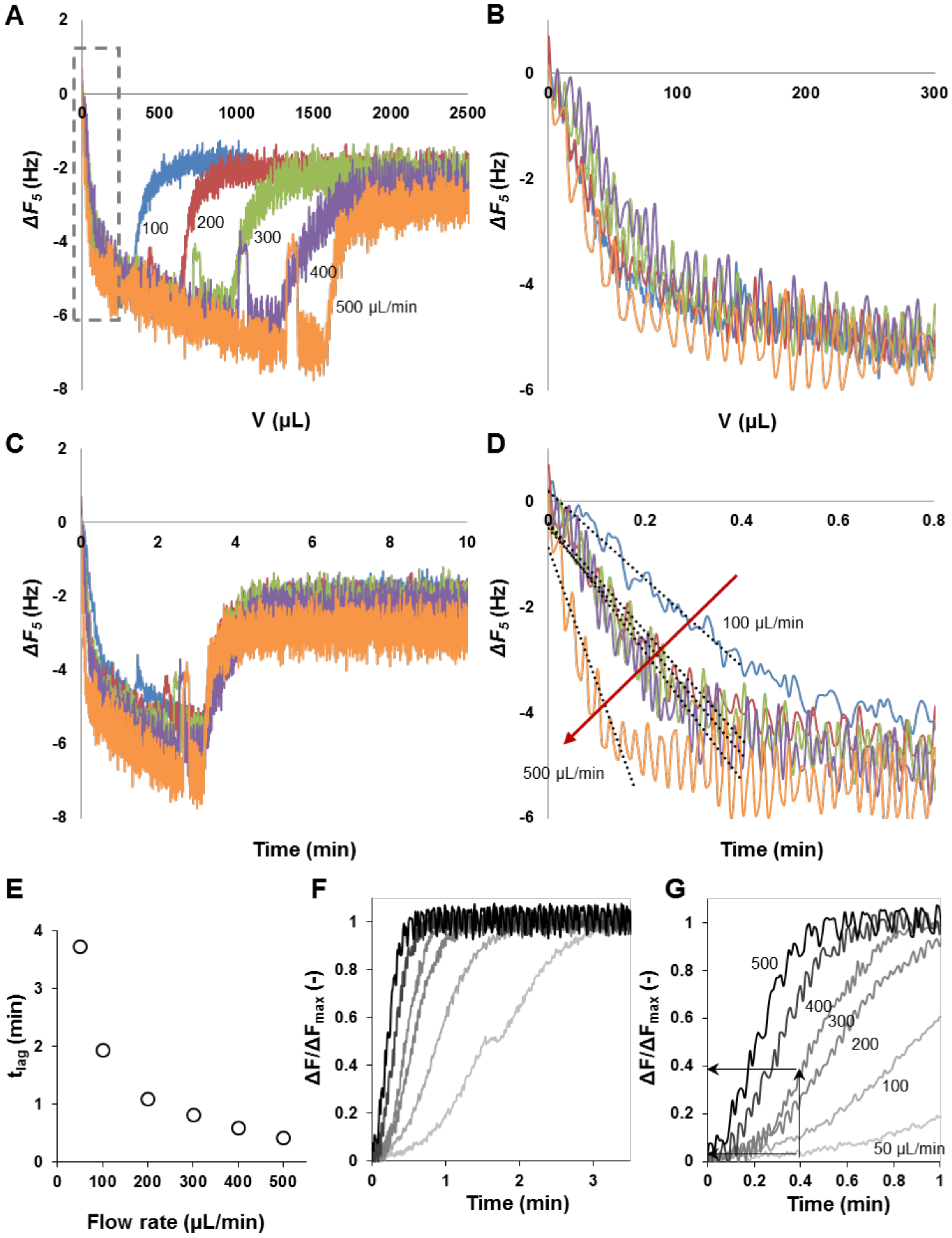

A
B
C
D
E
F
G
ΔF5 (Hz)
V (μL)
Time (min)
100
200
300
400
500 μL/min
100 μL/min
t_{lag} (min)
Flow rate (μL/min)
ΔF/ΔFmax (-)
50 μL/min
