# Supplementary material for: Interactions of nuclear transport factors and surface-conjugated FG nucleoporins: Insights and limitations
Source: PLoS One. 2019 Jun 6;14(6):e0217897. doi: 10.1371/journal.pone.0217897 (PMC6553764; doi:10.1371/journal.pone.0217897)

S2 Fig. QCM-D - Comparison of gold and silica sensor.

Nsp1FG was conjugated via its C-terminal cysteine to silica or gold surface without (A, C) or with mPEG passivation (B, D), respectively. The data shown are from 15 min binding-unbinding experiments to test the inertness of the two different surfaces. 1 µM Kap95 was used in all experiments.

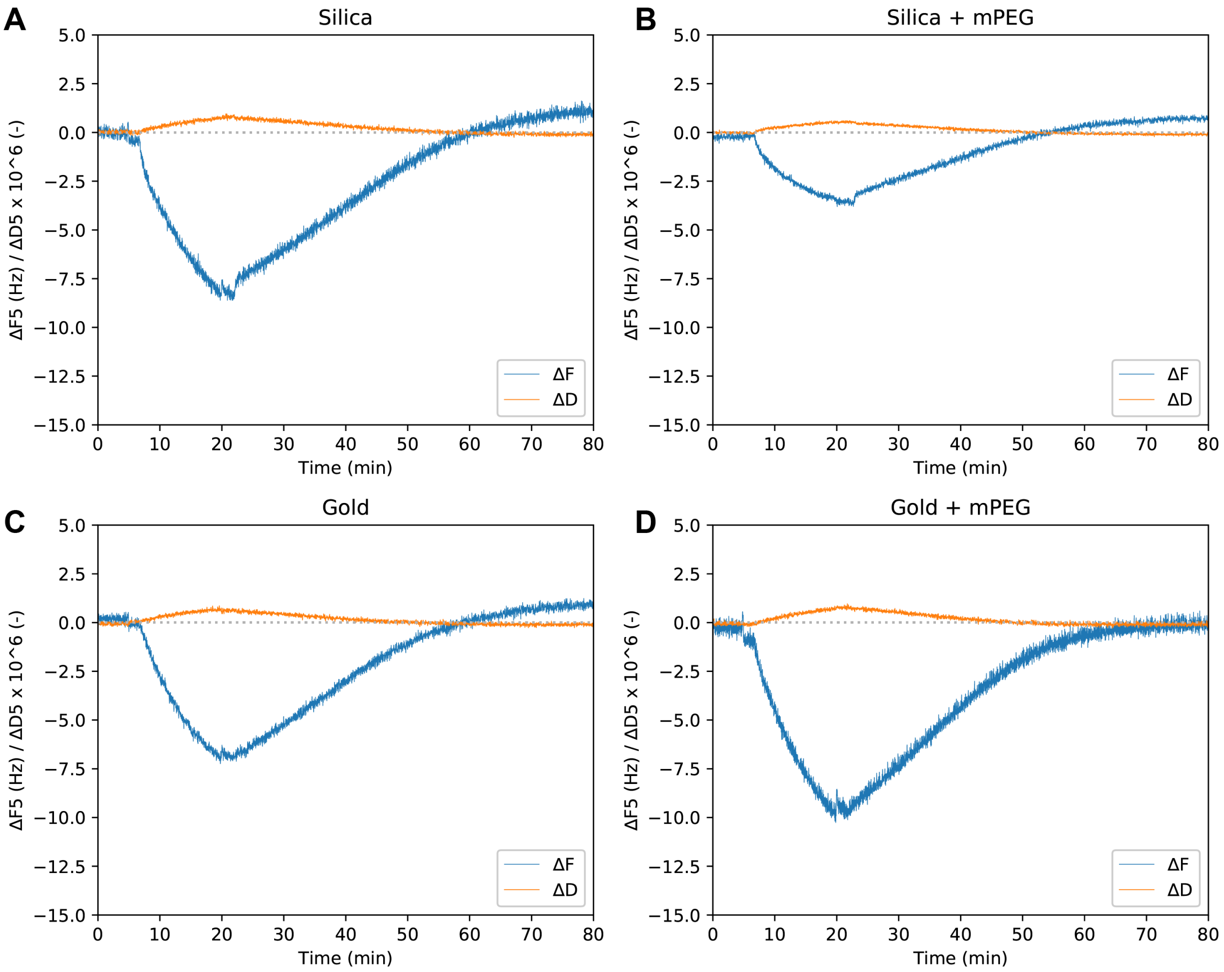

Supplement: S2 Fig — (PDF) [file pone.0217897.s005.pdf]
