# Supplementary material for: Interactions of nuclear transport factors and surface-conjugated FG nucleoporins: Insights and limitations
Source: PLoS One. 2019 Jun 6;14(6):e0217897. doi: 10.1371/journal.pone.0217897 (PMC6553764; doi:10.1371/journal.pone.0217897)

S3 Fig. QCM-D - Comparison of PEG passivators.

Gold sensors were modified with 2 mM solutions of (A) HS-$(CH_2)_{11}$-EG_3-OCH_3 ($mPEG_3$), (B) HS-$(CH_2)_{11}$-EG_3-OH ($hPEG_3$), and (C) HS-$(CH_2)_{11}$-EG_6-OH ($hPEG_6$) in ethanol, overnight and at room temperature. The data show a 30 min binding-unbinding experiment using a 300 μM BSA solution, in order to test the inertness of the different passivated surfaces. ΔF and ΔD indicate the change in resonance frequency and dissipation for the 5th overtone, respectively.

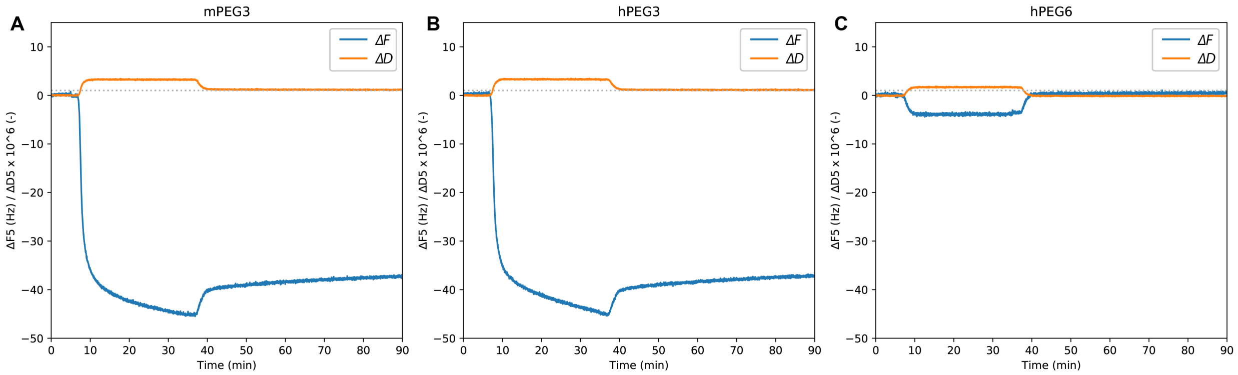

Supplement: S3 Fig — (PDF) [file pone.0217897.s006.pdf]
