# Supplementary material for: Interactions of nuclear transport factors and surface-conjugated FG nucleoporins: Insights and limitations
Source: PLoS One. 2019 Jun 6;14(6):e0217897. doi: 10.1371/journal.pone.0217897 (PMC6553764; doi:10.1371/journal.pone.0217897)
Supplement: S4 Fig — (PDF) [file pone.0217897.s007.pdf]

S4 Fig. QCM-D - Surface regeneration.

(A) Two silica sensors were modified with mPEG (upper) and Nsp1FG (lower) and then tested with GST-Kap95. The data show 6 experiments performed serially, varying binding time and buffer condition: (i) 2 min in TB-D, (ii) 2 min in TBS-D, (iii) 2 min in TBT-D, (iv) 60 min in TB-D, (v) 60 min in TBS-D, (vi) 60 min in TBT-D. A HUT wash step was included between each experiment to regenerate the surface. (B) Magnified views of the HUT washing steps in (A). (C) Multiple 1μM Kap95 binding experiments were repeated on the same Nsp1FG sensor. Regeneration with HUT solution and reproducibility of Kap95 binding was tested. Each color indicates replicate binding events with surface regenerations in between. F5 and D5 indicate 5th overtone of ΔF and ΔD, respectively. TB: 20 mM HEPES-KOH (pH 7.4), 110 mM KOAc, 2 mM $MgCl_2$, 10 μM $CaCl_2$, and 10 μM $ZnCl_2$; TB-D: TB with 5 mM DTT; TBS-D: TB-D with 150 mM NaCl; TBT: TB with 0.1% v/v Tween20; TBT-D: TBT with 5 mM DTT; TBT-PVP: TBT with 0.3% (w/v) polyvinylpyrrolidone. HUT: 50 mM HEPES, 8 M urea and 0.5% Tween20.

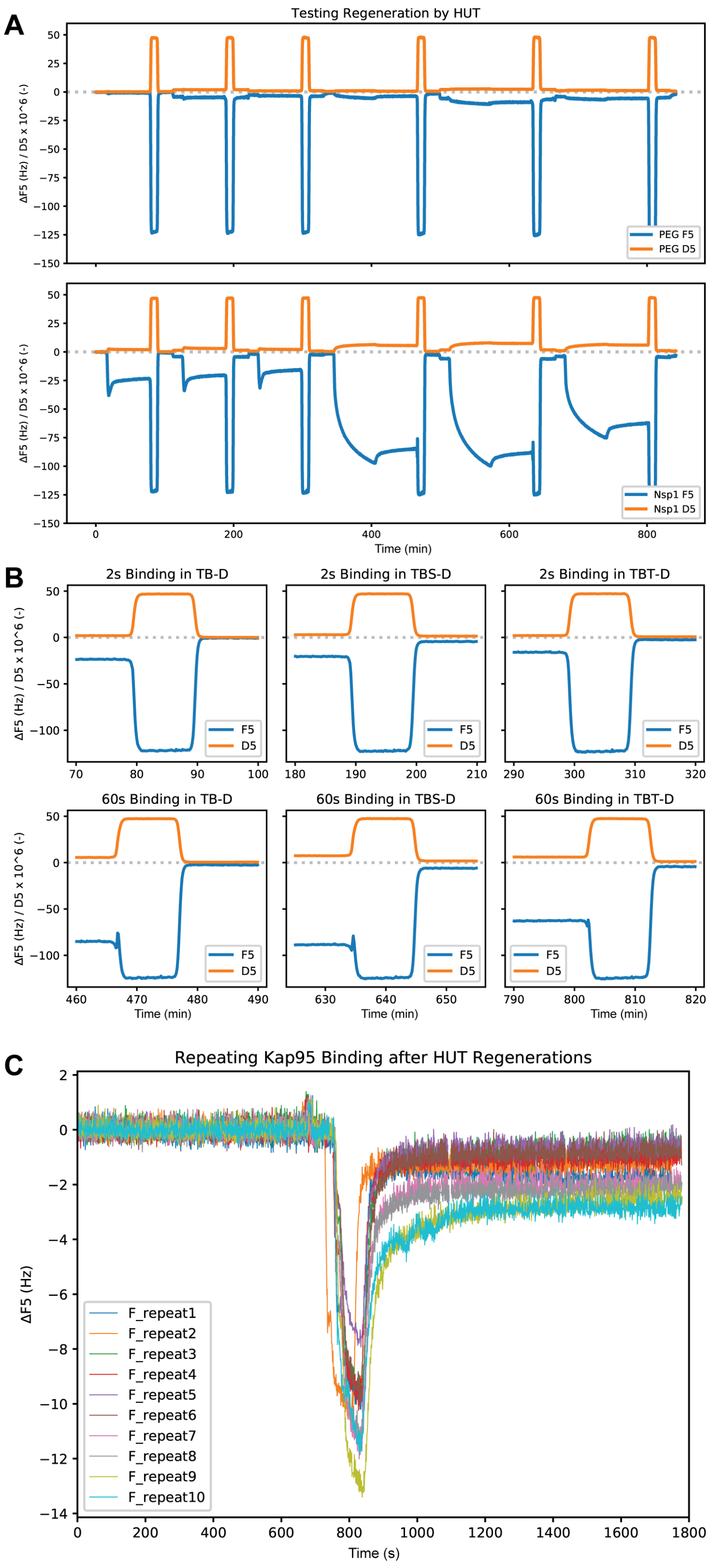
A
Testing Regeneration by HUT
ΔF5 (Hz) / D5 x 10^6 (-)
PEG F5
PEG D5
Nsp1 F5
Nsp1 D5
Time (min)
B
2s Binding in TB-D
2s Binding in TBS-D
2s Binding in TBT-D
60s Binding in TB-D
60s Binding in TBS-D
60s Binding in TBT-D
F5
D5
C
Repeating Kap95 Binding after HUT Regenerations
ΔF5 (Hz)
F_repeat1
F_repeat2
F_repeat3
F_repeat4
F_repeat5
F_repeat6
F_repeat7
F_repeat8
F_repeat9
F_repeat10
Time (s)
