# Supplementary material for: Interactions of nuclear transport factors and surface-conjugated FG nucleoporins: Insights and limitations
Source: PLoS One. 2019 Jun 6;14(6):e0217897. doi: 10.1371/journal.pone.0217897 (PMC6553764; doi:10.1371/journal.pone.0217897)

S5 Fig. QCM-D - Different harmonics during Nsp1FG conjugation.

The changes in frequency (ΔF) and energy dissipation (ΔD) for several overtones (3rd, 5th, 7th, 9th, and 11th) recorded in real time during Nsp1FG conjugation are shown. The Nsp1FG conjugation (60 min + 10 min buffer wash) was followed by a HUT wash (5 min), two passivation steps with mPEG (60 + 10 min buffer wash) and beta-mercaptoethanol (βME) (60 + 10 min buffer wash), and a final HUT wash (5 min). The spreading of the different harmonics indicates the viscoelastic nature of the Nsp1FG layer coupled to the sensor surface.

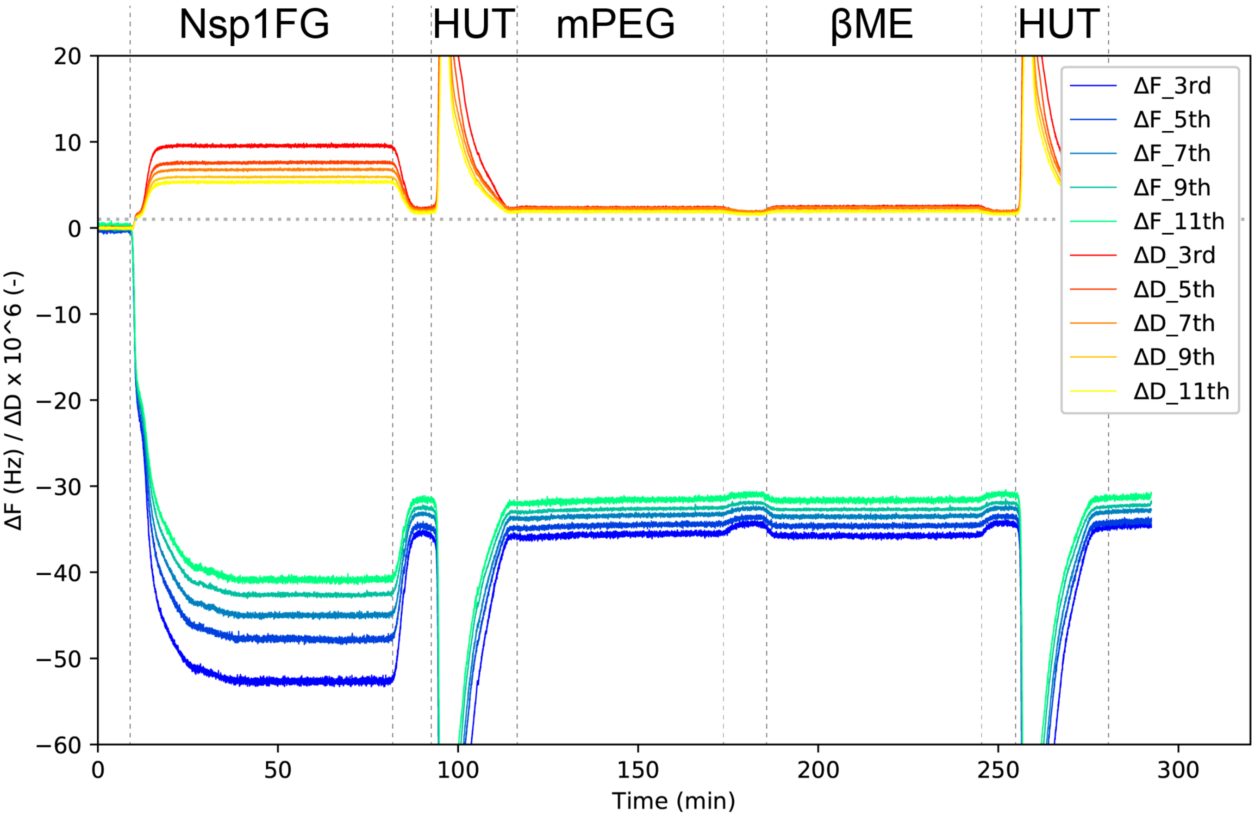

Supplement: S5 Fig — (PDF) [file pone.0217897.s008.pdf]
