# Supplementary material for: Interactions of nuclear transport factors and surface-conjugated FG nucleoporins: Insights and limitations
Source: PLoS One. 2019 Jun 6;14(6):e0217897. doi: 10.1371/journal.pone.0217897 (PMC6553764; doi:10.1371/journal.pone.0217897)

S6 Fig. AFM (volume force mapping) - Surface passivation with $hPEG_6$-C_{11}-SH.

Single molecule force spectroscopy maps between a gold-coated AFM tip and gold surfaces (A) without any passivation and after passivation with hPEG for, (B) 5 or (C) 30 minutes. White pixels (as the ones selected by blue circles in A-C) indicate strong gold/gold interactions (lack of passivation), while dark pixels (as the ones selected by red circles in B-C) show small or no adhesion (~passivated surface). (A) Without passivation, strong non-specific interactions (i.e. few hundreds of pN) between the AFM tip and the substrate resulted during volume force mapping, as indicated by the numerous high adhesions events (i.e. white pixels). The non-homogeneous nature of this interaction indicated by the spread of light and dark pixels, corresponding to stronger and weaker interactions, respectively, was likely due to damage or contamination on the AFM cantilever tip during the scan. (B) A 5 minutes incubation of the gold substrate with $hPEG_6$-C_{11}-SH allowed almost complete passivation, resulting in a mostly (~90%) inert surface with weak or no adhesion between the AFM cantilever tip and the gold surface: The average adhesion force, F_{ad} was less than 5 pN. Sparse spots resulted in high adhesion events similar to the negative control, suggesting that bare gold was still available for subsequent Nsp1FG immobilization. (C) A 30 minutes $hPEG_6$-C_{11}-SH incubation did not significantly differ from the 5 minute $hPEG_6$-C_{11}-SH incubation, which was expected since complete passivation of the surface (i.e. formation of a self-assembled monolayer due to the 11 carbons in the alkane chain, C_{11}) requires few hours of incubation (see reference [61] in main text). Force-distance curves for (D) gold-gold adhesion and (E) no adhesion. (F) Histogram representation of F_{ad} for the 5 min sample SMFS-map showed almost complete passivation, with F_{ad} < 5 pN.

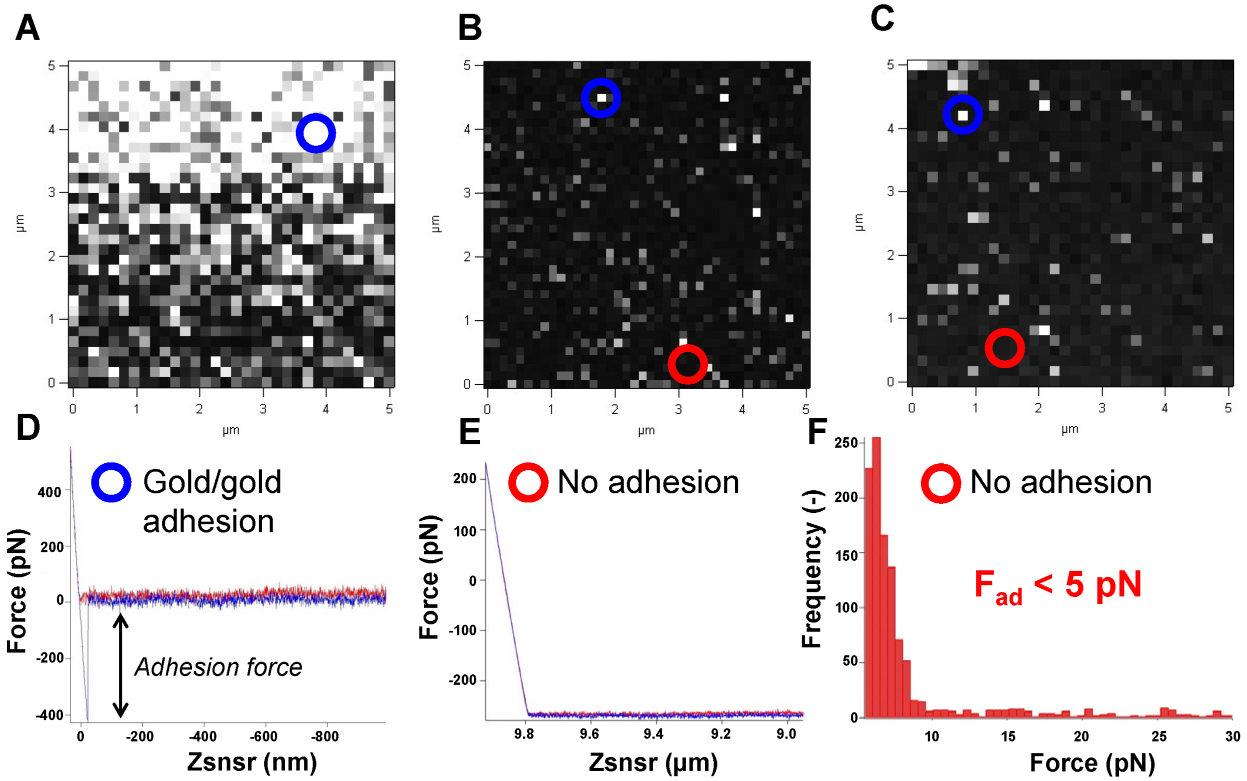

Supplement: S6 Fig — (PDF) [file pone.0217897.s009.pdf]
