# Supplementary material for: Interactions of nuclear transport factors and surface-conjugated FG nucleoporins: Insights and limitations
Source: PLoS One. 2019 Jun 6;14(6):e0217897. doi: 10.1371/journal.pone.0217897 (PMC6553764; doi:10.1371/journal.pone.0217897)

S7 Fig. AFM - Force-map of Nsp1FG for single molecule stretching.

(A) Single molecule force spectroscopy map of sNsp1 sample. Force-distance curves for (B) no adhesion (dark pixels in (A). An example indicated by a red circle) and (C) adhesion (white pixels in (A). An example indicated by a blue circle). Most of the surface exhibited no binding/adhesion to the AFM cantilever tips, as expected from the passivation. The adhesion events showed different characteristic patterns: (i) single-molecule stretching without an initial adhesion peak (74 out 1,600 ~5%) or (ii) with an initial adhesion peak (64 out 1,600 ~4%), (iii) multiple stretching events (20 out 1,600 ~1%) or (iv) non-reproducible surface/tip interactions (most likely due to non-specific/background interactions – data not used). A data set consisted of an array of 1,600 (40 x 40) force measurements, scanning in contact mode at 1 μm/s an area of 5 x 5 μm^2, with each pixel point spanning an approximate width of 125 nm in both X and Y. The trigger force was set to 500 pN.

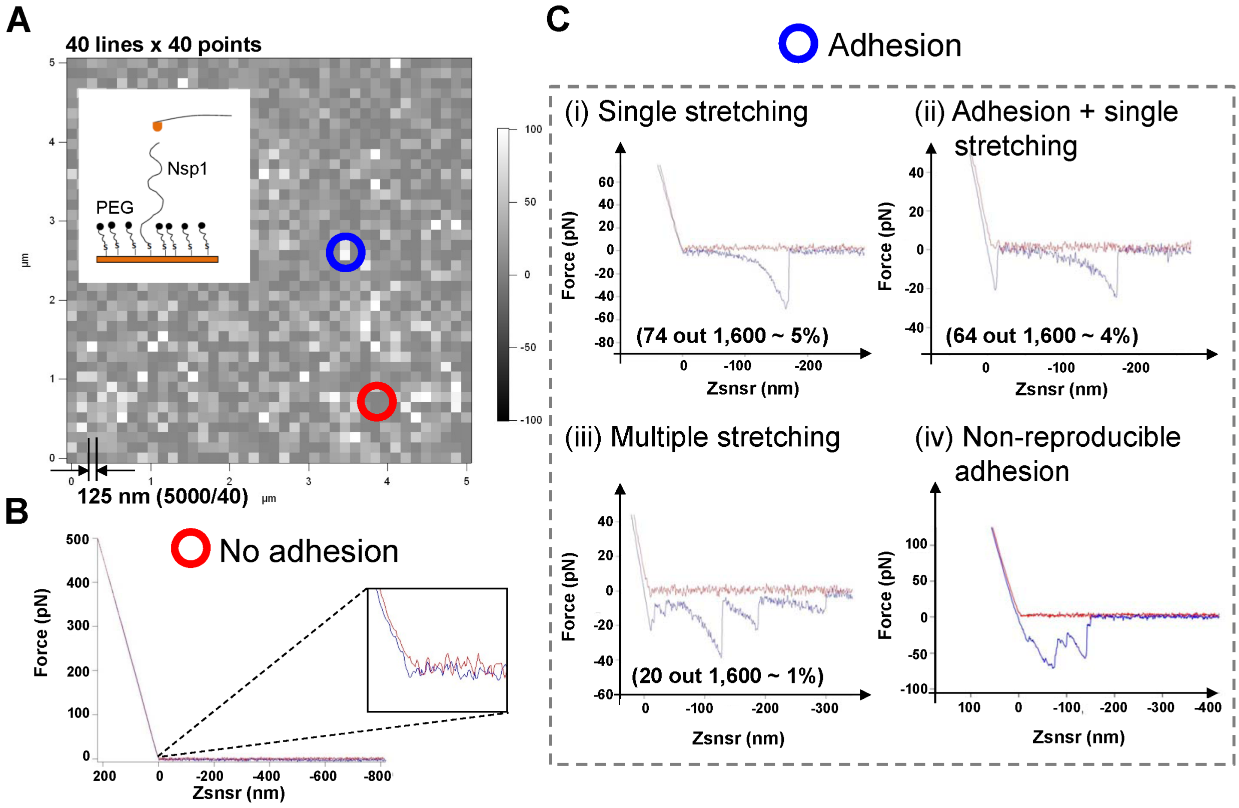

Supplement: S7 Fig — (PDF) [file pone.0217897.s010.pdf]
