# Supplementary material for: Interactions of nuclear transport factors and surface-conjugated FG nucleoporins: Insights and limitations
Source: PLoS One. 2019 Jun 6;14(6):e0217897. doi: 10.1371/journal.pone.0217897 (PMC6553764; doi:10.1371/journal.pone.0217897)

S8 Fig. AFM - Data fitting using the Worm Like Chain model.

Retraction curves during sNsp1 stretching in PBS are plotted as force, *F(x)*, versus sample-cantilever distance, *x*, and interpolated with the worm-like chain (WLC) model. Examples are reported for (A) single sNsp1 stretching with no initial adhesion peak (multiple runs are shown); (B) sNsp1 stretching with initial adhesion peak; and (C) multiple stretching events. The fitting line from the WLC model is also reported.

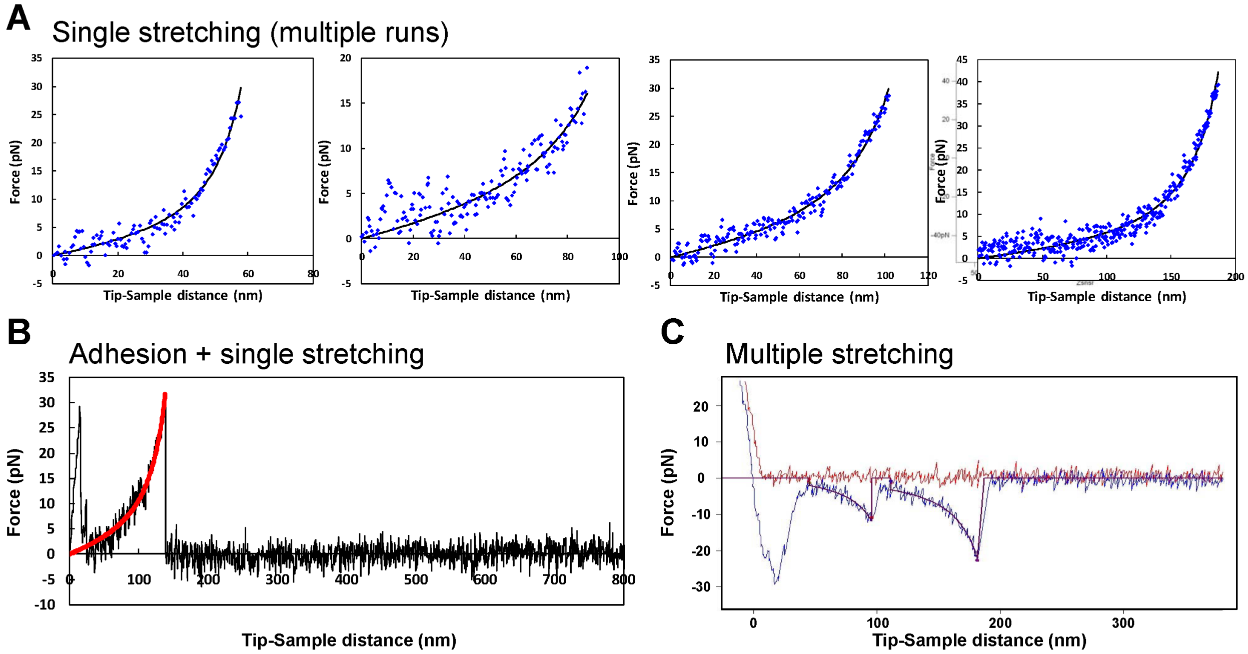

Supplement: S8 Fig — (PDF) [file pone.0217897.s011.pdf]
