# Supplementary material for: Interactions of nuclear transport factors and surface-conjugated FG nucleoporins: Insights and limitations
Source: PLoS One. 2019 Jun 6;14(6):e0217897. doi: 10.1371/journal.pone.0217897 (PMC6553764; doi:10.1371/journal.pone.0217897)

S9 Fig. AFM - Nsp1FG pulling experiment in TBT buffer.

Plots of the WLC model fitting parameters persistence length, L_p (blue), and contour length, L_C (green), versus the adhesion force, F_{ad}, from pulling sNsp1 at 1 μm/s in TBT buffer. Histograms (grey) represent the frequencies of L_p, L_C and F_{ad}. In the L_p plot, the average (solid line) ± 1 standard deviation (dash line) are also reported.

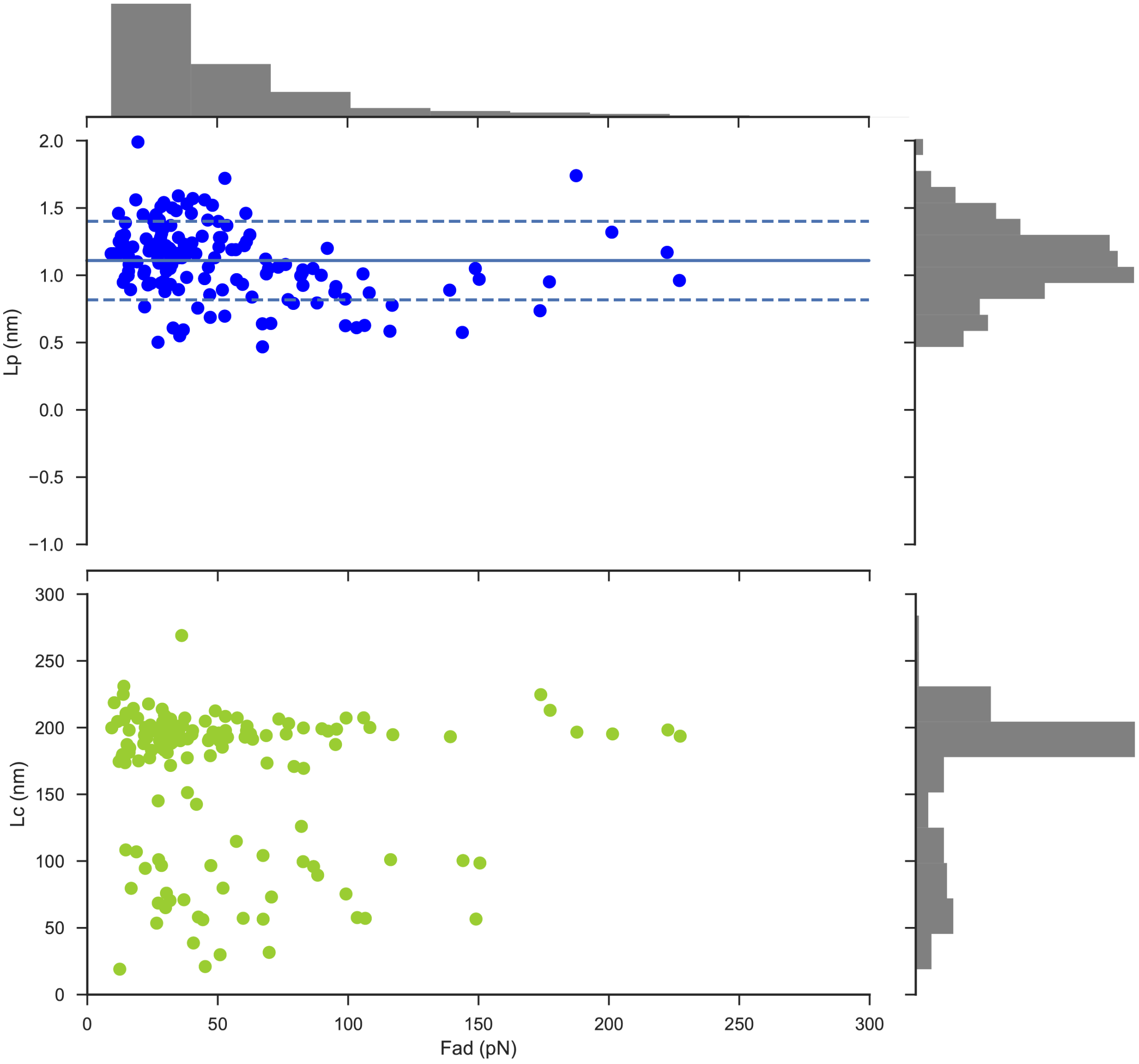

Supplement: S9 Fig — (PDF) [file pone.0217897.s012.pdf]
