# Supplementary material for: Interactions of nuclear transport factors and surface-conjugated FG nucleoporins: Insights and limitations
Source: PLoS One. 2019 Jun 6;14(6):e0217897. doi: 10.1371/journal.pone.0217897 (PMC6553764; doi:10.1371/journal.pone.0217897)

S10 Fig. AFM - Stretching Nsp1 in PBS at different pulling rates.

Plots of the WLC model fitting parameters persistence length, L_p (blue, top row), and contour length, L_C (green, bottom row), versus the adhesion force, F_{ad}, from pulling sNsp1 at (A) 3 µm/s, (B) 2 µm/s and (C) 1 µm/s in PBS buffer. Histograms (grey) represent the frequencies of L_p, L_C and F_{ad}. In the L_p plots, the average (solid line) ± 1 standard deviation (dash line) are also reported.

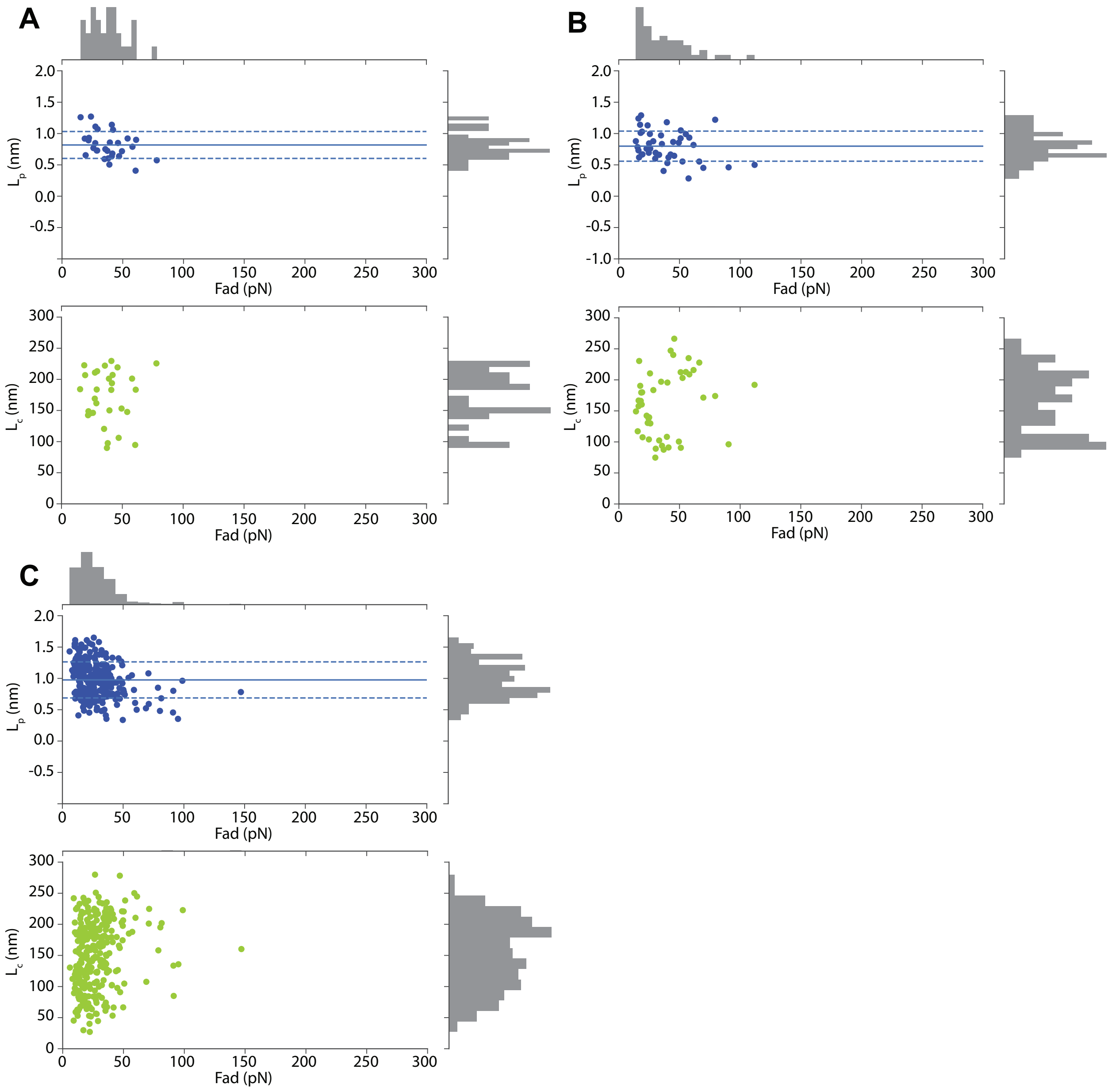

Supplement: S10 Fig — (PDF) [file pone.0217897.s013.pdf]
