# Supplementary material for: Interactions of nuclear transport factors and surface-conjugated FG nucleoporins: Insights and limitations
Source: PLoS One. 2019 Jun 6;14(6):e0217897. doi: 10.1371/journal.pone.0217897 (PMC6553764; doi:10.1371/journal.pone.0217897)

S11 Fig. QCM-D - GST-Kap95 binding with varying binding time.

The data below show 10 s, 30 s, 1 min, 2 min, and 60 min binding of 1 μM GST-Kap95 on an Nsp1FG-modified sensor, followed by a dissociation phase. The curvature of the binding curves indicates that equilibrium was never reached even after 60 min of binding time. ΔF and ΔD indicate the change in resonance frequency and dissipation for the 5th overtone, respectively.

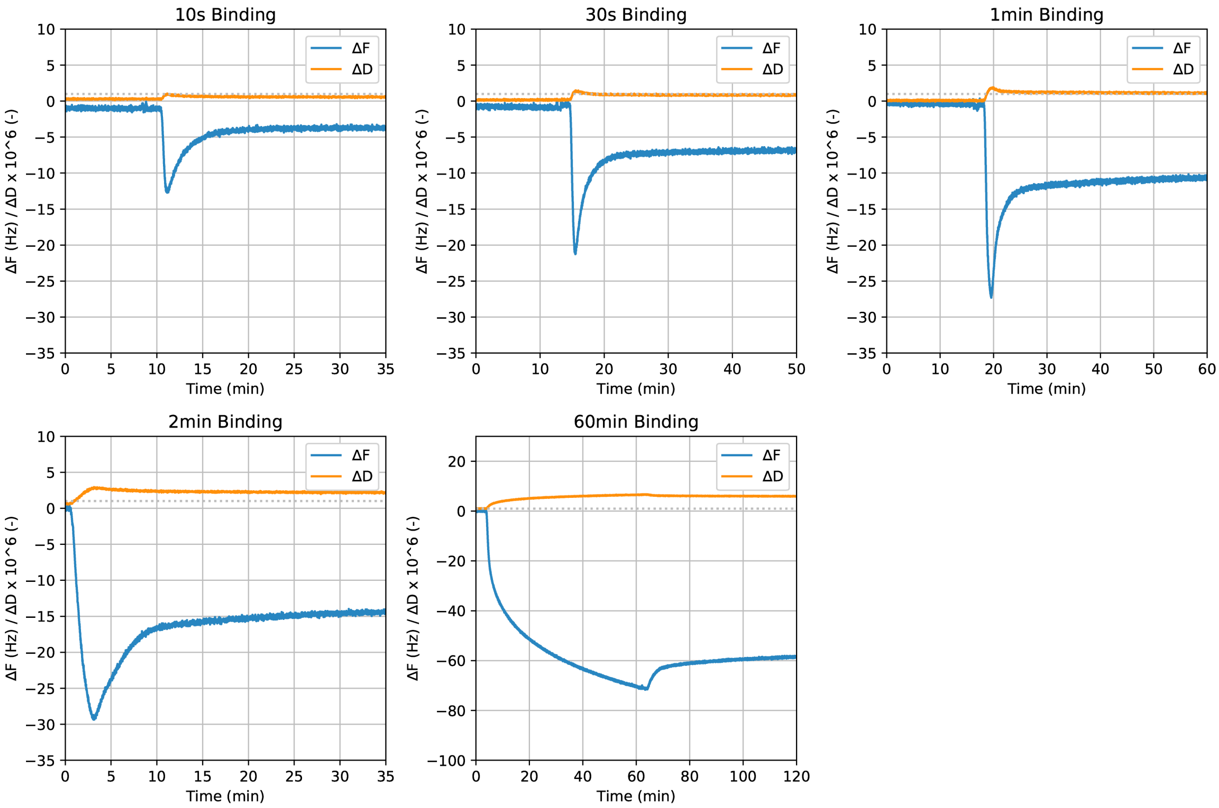

Supplement: S11 Fig — (PDF) [file pone.0217897.s014.pdf]
