# Supplementary material for: Interactions of nuclear transport factors and surface-conjugated FG nucleoporins: Insights and limitations
Source: PLoS One. 2019 Jun 6;14(6):e0217897. doi: 10.1371/journal.pone.0217897 (PMC6553764; doi:10.1371/journal.pone.0217897)
Supplement: S12 Fig — (PDF) [file pone.0217897.s015.pdf]

S12 Fig. SPR - Non-specific binding on a bare surface passivated with beta-mercaptoethanol.

Kap95 bindings on $FSFG_6$ (A-D) and a bare SPR surface passivated with beta-mercaptoethanol (E-H) are shown. Different rows indicate different lengths of the association phase: (A, E) 15 s, (B, F) 30 s, (C, G) 60 s, and (D, H) 120 s. While the SPR response on $FSFG_6$ surface was dependent on Kap95 concentration, it was not for the unconjugated, plain surface. The response came down to zero upon the onset of dissociation phase, suggesting that the plain surface was inert to Kap95.

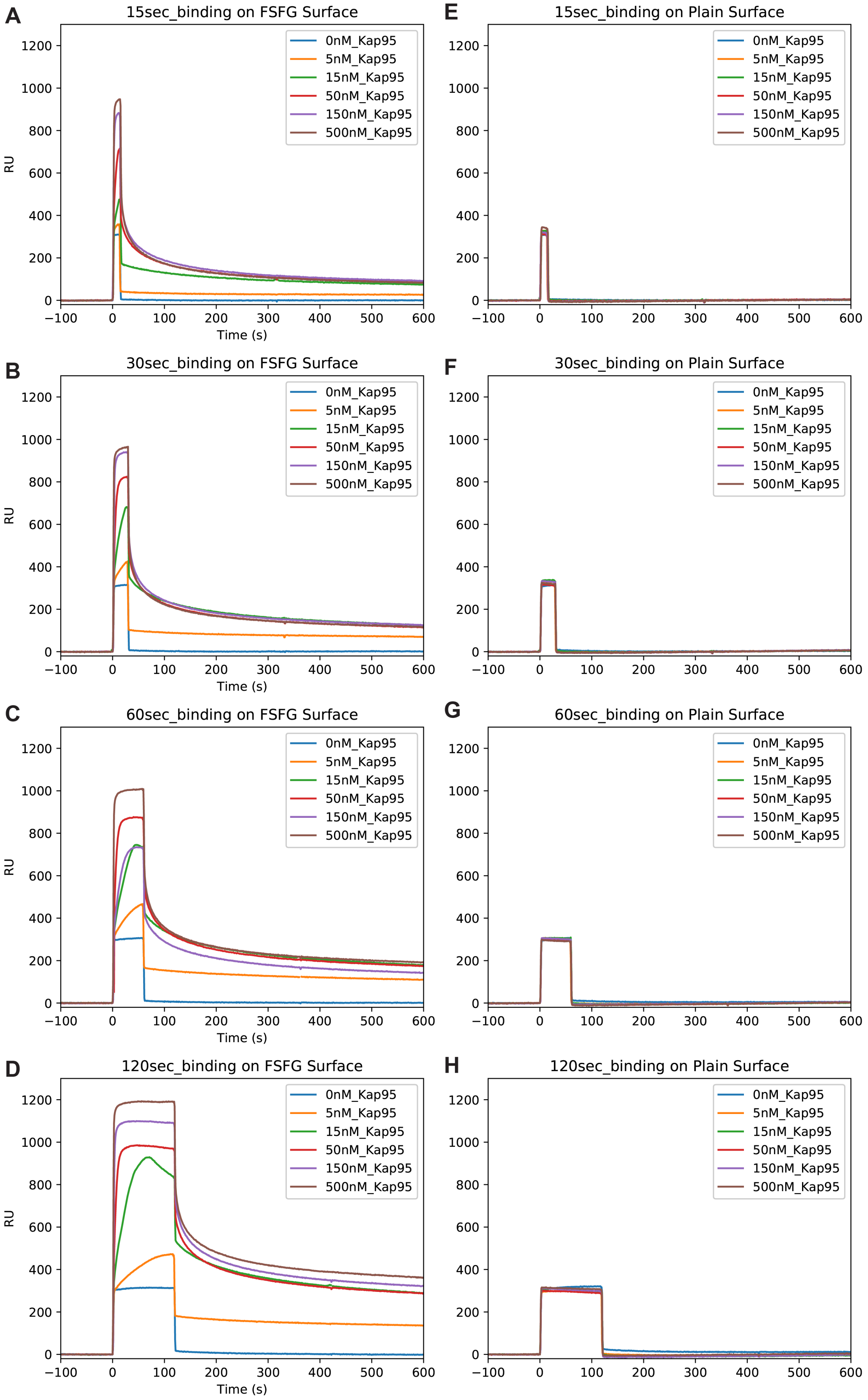
A
15sec_binding on FSFG Surface
E
15sec_binding on Plain Surface
B
30sec_binding on FSFG Surface
F
30sec_binding on Plain Surface
C
60sec_binding on FSFG Surface
G
60sec_binding on Plain Surface
D
120sec_binding on FSFG Surface
H
120sec_binding on Plain Surface
0nM_Kap95
5nM_Kap95
15nM_Kap95
50nM_Kap95
150nM_Kap95
500nM_Kap95
RU
Time (s)
0
200
400
600
800
1000
1200
−100
0
100
200
300
400
500
600
