# Supplementary material for: Interactions of nuclear transport factors and surface-conjugated FG nucleoporins: Insights and limitations
Source: PLoS One. 2019 Jun 6;14(6):e0217897. doi: 10.1371/journal.pone.0217897 (PMC6553764; doi:10.1371/journal.pone.0217897)

S13 Fig. SPR - Kap95 binding experiments.

Both association and dissociation phases of the Kap95 binding experiments are shown below. Rows indicate different $FSFG_6$ densities on the surface, and the columns indicate lengths of the association phase. The analysis of the dissociation phase from these experiments is shown in Fig. 4B.

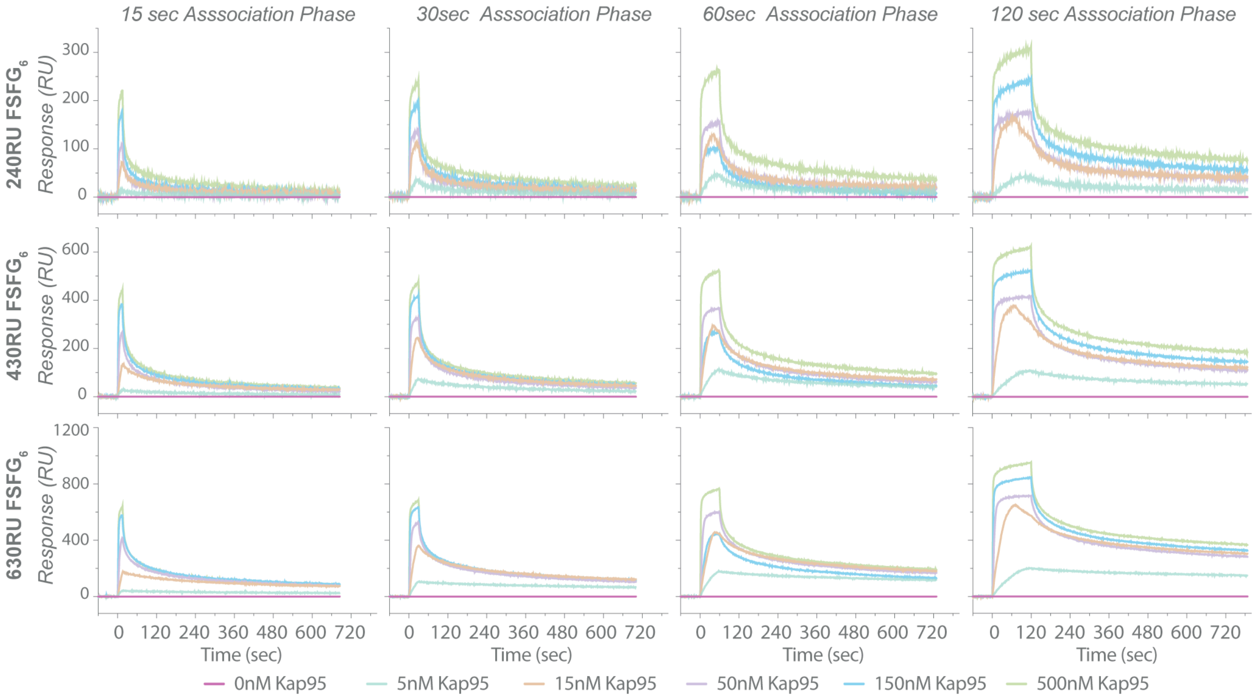

Supplement: S13 Fig — (PDF) [file pone.0217897.s016.pdf]
