# Supplementary material for: Interactions of nuclear transport factors and surface-conjugated FG nucleoporins: Insights and limitations
Source: PLoS One. 2019 Jun 6;14(6):e0217897. doi: 10.1371/journal.pone.0217897 (PMC6553764; doi:10.1371/journal.pone.0217897)
Supplement: S14 Fig — (PDF) [file pone.0217897.s017.pdf]

S14 Fig. SPR - Numerical simulation of SPR binding curves.

Build-up of analyte concentration in an SPR chamber was simulated under different conditions (S1 Text and S1 Table). (A-B) Comparison of analyte build-up in the presence and absence of surface ligand – i.e. (+) and (-) adsorption (ads). (A) It takes ~2-3 s to fully replace the SPR chamber volume. (B) Expansion of the first 0.5 s of (A) shows an additional mixing lag in the presence of surface ligand relative to the bare surface. Comparisons of (C-D) analyte build-up and (E-F) bound analyte at different flow rates. Even at flow rates that are an order of magnitude faster than those used in our SPR experiments (i.e., 100 µl/min) and with smaller model proteins that diffuse faster than those used in our experiments, the simulations indicate mixing lags approaching a few tenths of a second. This time scale is orders of magnitude larger than the time scale of FG-Kap95 interactions, suggesting that mass transport limitation was inevitable with our SPR setting. The reported values are averaged concentration values at the outlet of the SPR chamber for (A-D) and the total amount of analyte bound over the lower SPR reaction surface for (E-F) (see model description). (B), (D) and (F) are magnifications of (A), (C) and (E), respectively.

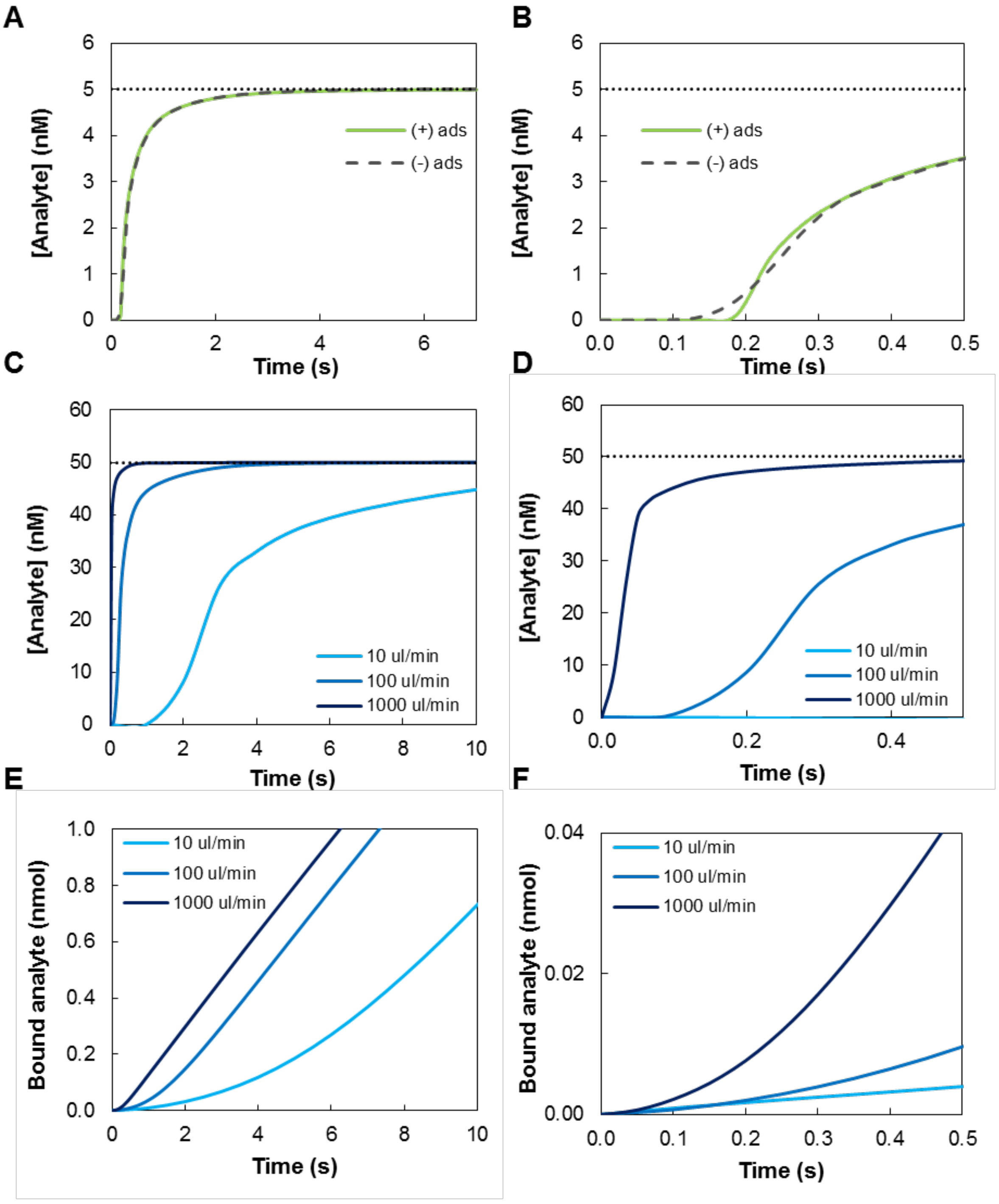

A
B
C
D
E
F
[Analyte] (nM)
Time (s)
(+) ads
(-) ads
10 ul/min
100 ul/min
1000 ul/min
Bound analyte (nmol)
