# Supplementary material for: Interactions of nuclear transport factors and surface-conjugated FG nucleoporins: Insights and limitations
Source: PLoS One. 2019 Jun 6;14(6):e0217897. doi: 10.1371/journal.pone.0217897 (PMC6553764; doi:10.1371/journal.pone.0217897)
Supplement: S15 Fig — (PDF) [file pone.0217897.s018.pdf]

S15 Fig. SPR - Fitting various kinetic models to binding curves.

SPR binding curves were fit to various kinetic models: one-to-one (A), one-to-one with MTL (B), two state models (C), heterogeneous ligand (D), and heterogeneous analyte (E) Binding curves on surfaces with different $FSFG_6$ densities are shown (columns (i) 425 RU, (ii) 550 RU, (iii) 791 RU). Data here are from a replicate experiment of the one shown in Fig. 4. Solid and dotted lines indicate the obtained sensorgrams and the respective fitted curves, respectively Kap95 concentrations are indicated in different colors. See S2 Table for the fitted parameters values.

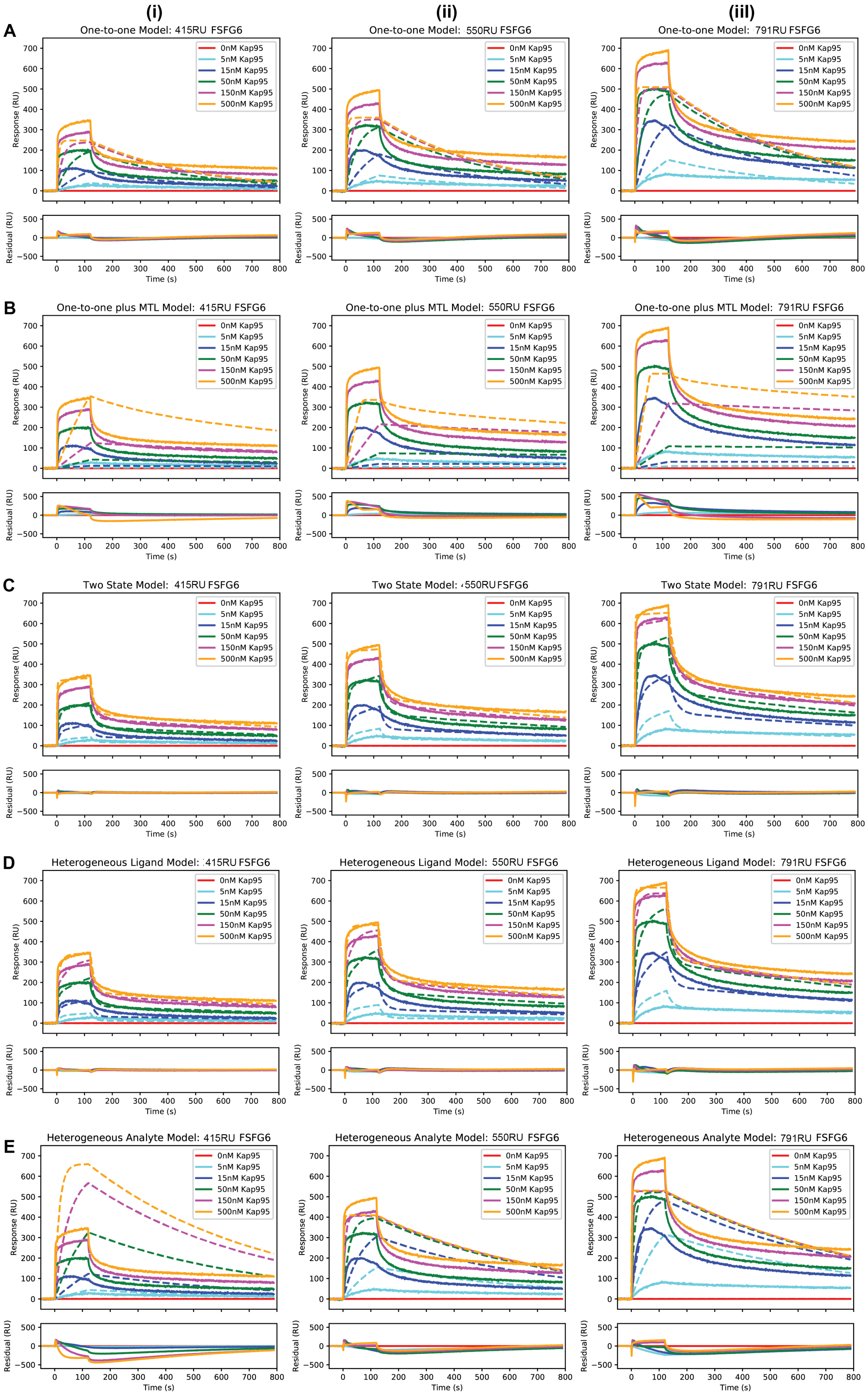

(i)
(ii)
(iii)
A
One-to-one Model: 415RU FSFG6
One-to-one Model: 550RU FSFG6
One-to-one Model: 791RU FSFG6
B
One-to-one plus MTL Model: 415RU FSFG6
One-to-one plus MTL Model: 550RU FSFG6
One-to-one plus MTL Model: 791RU FSFG6
C
Two State Model: 415RU FSFG6
Two State Model: 550RU FSFG6
Two State Model: 791RU FSFG6
D
Heterogeneous Ligand Model: 415RU FSFG6
Heterogeneous Ligand Model: 550RU FSFG6
Heterogeneous Ligand Model: 791RU FSFG6
E
Heterogeneous Analyte Model: 415RU FSFG6
Heterogeneous Analyte Model: 550RU FSFG6
Heterogeneous Analyte Model: 791RU FSFG6
0nM Kap95
5nM Kap95
15nM Kap95
50nM Kap95
150nM Kap95
500nM Kap95
Response (RU)
Residual (RU)
Time (s)
