# Supplementary material for: Interactions of nuclear transport factors and surface-conjugated FG nucleoporins: Insights and limitations
Source: PLoS One. 2019 Jun 6;14(6):e0217897. doi: 10.1371/journal.pone.0217897 (PMC6553764; doi:10.1371/journal.pone.0217897)

S16 Fig. QCM-D - Conjugation of $FSFG_6$ and $SSSG_6$ to silica sensors.

The changes in frequency (ΔF) and energy dissipation (ΔD) for several overtones were recorded in real time during (A) $FSFG_6$ and (B) $SSSG_6$ conjugations. The overlapping of the different harmonics is indicative of the rigid nature of these layers. (C-D) Sauerbrey model was used to calculate the layer thickness, for different layer densities (1,100, 1,200 and 1,300 kg/m^3).

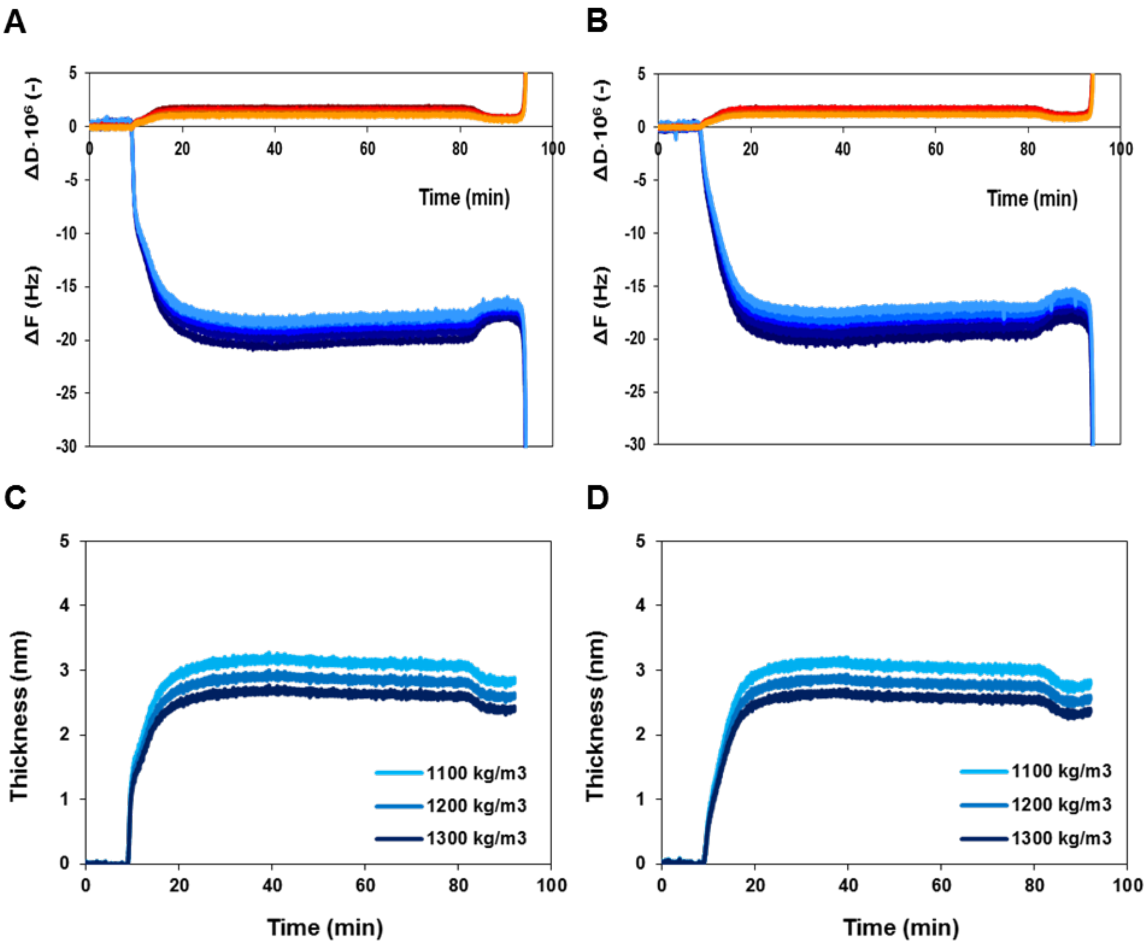

Supplement: S16 Fig — (PDF) [file pone.0217897.s019.pdf]
