# Supplementary material for: Interactions of nuclear transport factors and surface-conjugated FG nucleoporins: Insights and limitations
Source: PLoS One. 2019 Jun 6;14(6):e0217897. doi: 10.1371/journal.pone.0217897 (PMC6553764; doi:10.1371/journal.pone.0217897)
Supplement: S1 Table — (PDF) [file pone.0217897.s020.pdf]

S1 Table. SPR - Numerical simulation parameters.

Summary of the parameters used for the SPR numerical simulations in S13 Fig.

Parameter description	Value	Units
SPR geometry		
Channel depth, D	$1\cdot10^{-3}$	m
Channel width, W	$1\cdot10^{-3}$	m
Channel height, H	$5\cdot10^{-4}$	m
Chamber volume, D·W·H	$5\cdot10^{-10}$	m^3
Surface area for adsorption, D·W	$1\cdot10^{-6}$	m^2
Outlet surface area, D·H	$5\cdot10^{-7}$	m^2
Flow properties		
Inlet velocity[a]	$(0.3 – 3 – 30)\cdot10^{-3}$	m/s
H_2O concentration	55,000	mol/m^3
H_2O viscosity	$8.9\cdot10^{-4}$	Pa·s
H_2O molecular weight	18	g/mol
H_2O density	1,000	kg/m^3
Temperature	298	K
Ligand		
Molecular weight, MW_L	28,771	g/mol
Experimental RU[b]	500 - 1,500	-
Total binding sites[c]	$(RU/1,000)/MW_L$	mol/m^2
Analyte		
Molecular weight, MW_A	15,565	g/mol
Inlet concentration	$5\cdot10^{-5}$	mol/m^3
Binding		
Adsorption rate constant	$2\cdot10^{2}$	$mol/(m^3\cdot s)$
Desorption rate constant	$2\cdot10^{-3}$	1/s

[a] The feed velocity was 3 mm/s, corresponding to the experimental 100 µL/min, for S13A-B Fig, while it was varied from 0.3 to 30 mm/s for S13C-F Fig.

[b] Experimental SPR response unit (RU) signal was 1,500 (i.e. high ligand density) for S13A-B Fig and 500 (i.e. medium ligand density) for S13C-F Fig.

[c] It was calculated using the correlation 1 RU = 1,000 ng/mm^2.
