# Supplementary material for: Interactions of nuclear transport factors and surface-conjugated FG nucleoporins: Insights and limitations
Source: PLoS One. 2019 Jun 6;14(6):e0217897. doi: 10.1371/journal.pone.0217897 (PMC6553764; doi:10.1371/journal.pone.0217897)
Supplement: S2 Table — (PDF) [file pone.0217897.s021.pdf]

S2 Table. SPR - Fitting kinetic and equilibrium models to binding curves

SPR binding curves (solid lines in S14 Fig) were fit to various kinetic models: one-to-one, one-to-one with MTL, heterogeneous ligands, heterogeneous analytes, and two state models. Langmuir isotherm analysis was also included. FG density refers to the amount of $FSFG_6$ conjugated on the SPR sensors: low (415 RU), medium (550 RU) and high (791 RU). Binding time indicate the length of the association phase: 15, 30, 60 and 120 seconds.

One-to-one model

FG Density	Binding Time (s)	ka (10^5 1/Ms)	kd (10^{-3} 1/s)	KD (10^{-9} M)	R_{max} (RU)	Chi^2 (RU)
Low	15	10.80	3.70	3.42	142.4	516
	30	5.32	3.01	5.65	182.6	711
	60	5.49	3.01	5.49	218.0	1173
	120	3.21	2.61	8.14	251.5	1154
Medium	15	20.70	3.03	1.46	222.4	974
	30	8.10	2.65	3.27	279.8	1403
	60	8.73	2.75	3.15	319.5	2349
	120	4.59	2.50	5.45	362.8	2383
High	15	24.10	2.09	0.87	386.7	1643
	30	9.97	1.99	1.99	442.5	2364
	60	12.9	2.20	1.70	470.7	4077
	120	6.82	2.19	3.20	512.5	4249

One-to-one plus mass transport limitation model

FG Density	Binding Time (s)	ka (10^5 1/Ms)	kd (10^{-3} 1/s)	KD (10^{-9} M)	R_{max} (RU)	kt (10^4 RU/Ms)	Chi^2 (RU)
Low	15	9.75	6.53	6.70	208.0	1	2129
	30	42.90	4.49	1.05	74.6	991	1642
	60	9.05	4.09	4.52	227.6	877	2973
	120	1.15	3.26	28.30	434.2	849	5035
Medium	15	25.60	4.65	1.82	166.8	1	6896
	30	44.50	3.30	0.74	99.0	1560	5199
	60	15.50	3.86	2.49	271.2	1210	7340
	120	8.51	3.18	3.74	338.2	1290	7949
High	15	3.58	3.35	9.36	754.9	1	27110
	30	35.70	2.70	0.76	127.6	3850	17795
	60	28.30	3.14	1.11	315.9	1840	21187
	120	16.60	2.75	1.65	466.0	1830	20928

Heterogeneous analytes model

FG Density	Binding Time (s)	ka ($10^5$1/Ms)	kd (10^{-3} 1/s)	KD (10^{-9} M)	R_{max} (RU)	ka_2 ($10^5$1/Ms)	kd_2 (10^{-3} 1/s)	KD_2 (10^{-9} M)	Chi^2 (RU)
Low	15	1.67	3.27	19.60	515.0	1.67	3.27	19.60	6092
	30	21.9	2.24	1.03	80.5	21.90	2.24	1.03	1341
	60	4.66	2.04	4.38	282.1	4.66	2.04	4.38	3784
	120	0.612	1.63	26.60	677.2	0.61	1.63	26.60	27094
Medium	15	12.3	2.33	1.89	258.3	12.30	2.33	1.89	1710
	30	22.5	1.65	0.73	107.4	22.50	1.65	0.73	3789
	60	7.89	1.93	2.45	363.5	7.89	1.93	2.45	5473
	120	4.36	1.59	3.65	411.3	4.36	1.59	3.65	7967
High	15	4.63	1.68	3.62	641.9	4.63	1.68	3.62	10615
	30	18.8	1.35	0.72	157.8	18.80	1.35	0.72	12260
	60	14.9	1.57	1.05	380.1	14.90	1.57	1.05	5768
	120	8.4	1.37	1.64	530.4	8.40	1.37	1.64	13870

Heterogeneous ligands model

FG Density	Binding Time (s)	ka ($10^5$1/Ms)	kd (10^{-3} 1/s)	KD (10^{-9} M)	R_{max} (RU)	ka_2 ($10^5$1/Ms)	kd_2 (10^{-3} 1/s)	KD_2 (10^{-9} M)	Chi^2 (RU)
Low	15	31.90	85.4	26.70	243.0	4.27	1.19	2.77	83
	30	20.10	80.2	39.90	251.9	2.61	1.00	3.84	87
	60	38.90	82.7	21.30	228.7	1.95	0.87	4.43	159
	120	1.38	0.7	5.12	152.9	33.20	71.90	21.70	125
Medium	15	20.10	1.5	0.76	142.6	20.50	74.90	36.50	222
	30	5.72	1.1	1.99	183.2	18.30	68.20	37.30	248
	60	4.48	1.0	2.14	198.5	39.50	68.80	17.40	467
	120	2.17	0.7	3.32	219.6	35.70	57.70	16.20	368
High	15	15.10	54.6	36.20	452.5	25.60	1.28	0.50	360
	30	9.39	1.0	1.09	317.6	14.50	57.00	39.30	373
	60	11.60	1.0	0.83	310.4	26.10	60.00	23.00	918
	120	4.86	0.8	1.60	321.8	31.90	54.10	17.00	823

Two state model

FG Density	Binding Time (s)	ka ($10^5$1/Ms)	kd (10^{-3} 1/s)	KD (10^{-9} M)	R_{max} (RU)	ka_2 (10^{-3} 1/s)	kd_2 (10^{-3} 1/s)	Chi^2 (RU)
Low	15	18.60	102.0	54.7	357.6	12.10	1.60	82
	30	11.80	85.5	72.4	384.4	9.91	1.25	75
	60	18.40	95.4	51.8	372.0	7.17	1.05	127
	120	14.70	78.6	53.3	352.7	5.24	0.86	78
Medium	15	28.20	55.7	19.8	453.6	12.80	1.80	194
	30	14.30	56.9	39.8	521.7	10.60	1.35	177
	60	22.20	64.0	28.8	508.4	7.42	1.09	316
	120	17.00	60.1	35.3	492.5	5.29	0.87	208
High	15	29.90	28.7	9.6	639.8	13.50	1.79	428
	30	15.40	33.4	21.7	716.3	11.20	1.28	369
	60	25.20	36.0	14.3	683.6	7.65	1.05	801
	120	19.30	39.5	20.5	667.4	5.40	0.84	523

Langmuir isotherm

FG Density	Binding Time (s)	KD (10^{-9} M)	R_{max} (RU)	Chi^2 (RU)
Low	15	64.2	382.9	218
	30	76.8	397.9	345
	60	43.3	375.5	326
	120	41.9	357.0	109
Medium	15	59.3	580.4	1293
	30	63.5	585.4	1382
	60	30.8	537.1	1042
	120	30.4	505.7	437
High	15	64.5	892.1	2203
	30	54.7	860.3	4405
	60	22.6	764.5	3062
	120	22.4	708.9	1519
